# Ictal-onset localization through effective connectivity analysis based on RNN-GC with intracranial EEG signals in patients with epilepsy

**DOI:** 10.1186/s40708-024-00233-y

**Published:** 2024-08-23

**Authors:** Xiaojia Wang, Yanchao Liu, Chunfeng Yang

**Affiliations:** 1 Wuxi Vocational College of Science and Technology, Wuxi, 214028 China; 2 School of computer science and engineering, Southeast University, Nanjing, 210096 China

**Keywords:** Recurrent neural network, Granger causality, Effect connectivity, Intracranial EEG signal

## Abstract

Epilepsy is one of the most common clinical diseases of the nervous system. The occurrence of epilepsy will bring many serious consequences, and some patients with epilepsy will develop drug-resistant epilepsy. Surgery is an effective means to treat this kind of patients, and lesion localization can provide a basis for surgery. The purpose of this study was to explore the functional types and connectivity evolution patterns of relevant regions of the brain during seizures. We used intracranial EEG signals from patients with epilepsy as the research object, and the method used was GRU-GC. The role of the corresponding area of each channel in the seizure process was determined by the introduction of group analysis. The importance of each area was analysed by introducing the betweenness centrality and PageRank centrality. The experimental results show that the classification method based on effective connectivity has high accuracy, and the role of the different regions of the brain could also change during the seizures. The relevant methods in this study have played an important role in preoperative assessment and revealing the functional evolution patterns of various relevant regions of the brain during seizures.

## Introduction

Epilepsy is one of the most common clinical diseases in the nervous system. It is a type of transient and repetitive syndrome characterized by central nervous system dysfunction, which is caused by the high synchronization and self limited abnormal discharge of brain neurons [1]. The occurrence of epilepsy can have serious consequences, including disrupting normal study and work, and increasing the risk of injury, depression and suicide. Due to the high incidence of epilepsy, its treatment has become a hot research topic in recent years.

Drug therapy is an effective way to alleviate seizures. After treatment with antiepileptic drugs, only 3–5% of patients get remission each year, while 71–80% of patients have epilepsy recurrence, and, for about 30% of patients who are drug-resistant, this pathology affects their life [2, 3]. For these drug-resistant epilepsy patients, surgical removal of the epileptogenic zone (EZ), which is defined as the key cortical region of clinical seizures [4, 5], should be considered [6]. The key to the success of surgical treatment is how to accurately locate the EZ and brain functional areas (such as movement, sensation, vision, language, and memory, etc.), through preoperative evaluation, so as to ensure that the patient’s various learning and cognition abilities after surgery are not affected too much. Intracerebral EEG (iEEG) is the gold standard for clinical diagnosis of epilepsy and provides support for preoperative evaluation of surgery. Its high temporal resolution allows it to accurately capture the rapid dynamics of brain activity. The connectivity analysis of iEEG signals can provide scientific basis for the early diagnosis of epilepsy and has important medical significance [7–9]. And effective connectivity analysis focuses on the direction of information flow between activated brain regions. Using the multi-channel iEEG signals to roughly identify the location of the lesion has become an effective method for the diagnosis of epilepsy [10].

In the past few years, there are a number of algorithms for evaluating effective cerebral connectivity. The Wiener-Granger Causality Index (WGCI) [11] is a linear autoregressive (AR) model based on a stochastic process. It can detect the direct causal relationship between two time series. With the deepening of WGCI method research, these theories have been generalized from bivariate to multivariate not only in the time domain [12] but also in the frequency domain [13]. In recent years, these methods have been extended to non-linear cases [14] and successfully applied to neuroscience [15–17]. Kamiński et al. proposed a Directed Transfer Function (DTF) method [18] to enable analysis of causality between multi-channel signals. Baccalá et al. proposed a clearer and more accurate frequency domain connection method based on Granger causality, called Partial Directed Coherence (PDC) [19]. When analyzing bivariate signals, PDC is equivalent to the DTF method, but in multivariate signal analysis, PDC can distinguish between direct and indirect causal links, which is more advantageous. However, PDC can only detect the causal relationship in the frequency domain of the linear model, and it is powerless for the nonlinear model PDC method. He Fei et al. proposed a nonlinear PDC (NPDC) [20] by modelling nonlinear relationships and using corresponding nonlinear frequency domain analysis techniques. The advantage of this method is that in the linear case, it is equivalent to the PDC method, but in the non-linear case, it can detect both linear causality and non-linear one.

Due to the rise of deep learning, Artificial Neural Networks (ANNs) have a certain driving role in all professions and trades. Raval et al. [21] pointed out that machine learning technology has been used in medical diagnosis to analyze diseases based on clinical and laboratory symptoms to provide accurate results. They also pointed out that ANNs are one of the main techniques for solving medical diagnostic problems. Montalto et al. [22] proposed the use of Neural Network-based Granger causality (NN-GC) to characterize the directed relationship between brain signals and obtain better performance. Although this method works well in non-linear situations, it usually requires longer stationary signals and is sensitive to noise [23]. WGCI, PDC, NPDC usually require a fixed order of the AR model, which means that the signal has a fixed propagation delay. This assumption contradicts the nature of brain information transmission. Because the cerebral information transmission pathways may be diverse, the signal transmission delay is uncertain, and there may even be long-distance transmission delays. To deal with these problems, Yueming et al. [24] proposed a Recurrent Neural Network-based Granger causality (RNN-GC) method for multivariate brain effective connectivity estimation. The RNN-GC model can take time series with arbitrary delays as input, and use Long Short-Term Memory (LSTM) model [25] to learn the information flow from the data. RNN-GC can learn from time lags of different lengths, which is effective even in very long transmission delays.

In this work, the objective is to use the RNN-GC method to perform effective connectivity analysis on real iEEG signals from patients with drug-resistant epilepsy. iEEG data was collected by implanting electrodes into the cerebral cortex of patients. The data of a certain channel of iEEG is the potential difference between two adjacent electrodes, and the electrodes correspond to different areas of the brain: onset area, propagation area, and not-involved area. According to the results of RNN-GC, group analysis is performed on the multi-channel iEEG data. Group analysis is an effective method for localization of lesions, and provides a certain basis for preoperative evaluation of surgery. In addition, we divided seizures into three phases (pre-ictal, ictal, post-ictal) to study the evolutionary pattern of effective connectivity over time. The distributed information transmission during the seizure can also be described by a network model, including a set of nodes (neurons, regions) and edges (interregional connections, pathways) [26]. This paper uses metrics such as betweenness centrality and PageRank centrality to reveal the importance of each node in the graph model of seizures, and provides a powerful basis for the localization of lesions.

## Materials and methods

### Dataset

Intracranial EEG signals were collected from multiple seizures of the same epileptic patient. The experiments in this paper used three real signals: seizure 1, seizure 2, and seizure 3. It corresponds to three seizures in patients with epilepsy. The seizure process of the three data records is relatively complete, and after preliminary analysis by clinical experts. The analysis of the data will have higher confidence. The detailed information of the data is shown in Table 1.

**Table 1:** Details of the datasets

Data	Sample frequency [Hz]	Length [s]	Channels
Seizure 1	256	72	20
Seizure 2	256	64	20
Seizure 3	256	64	20

**Fig. 1:**
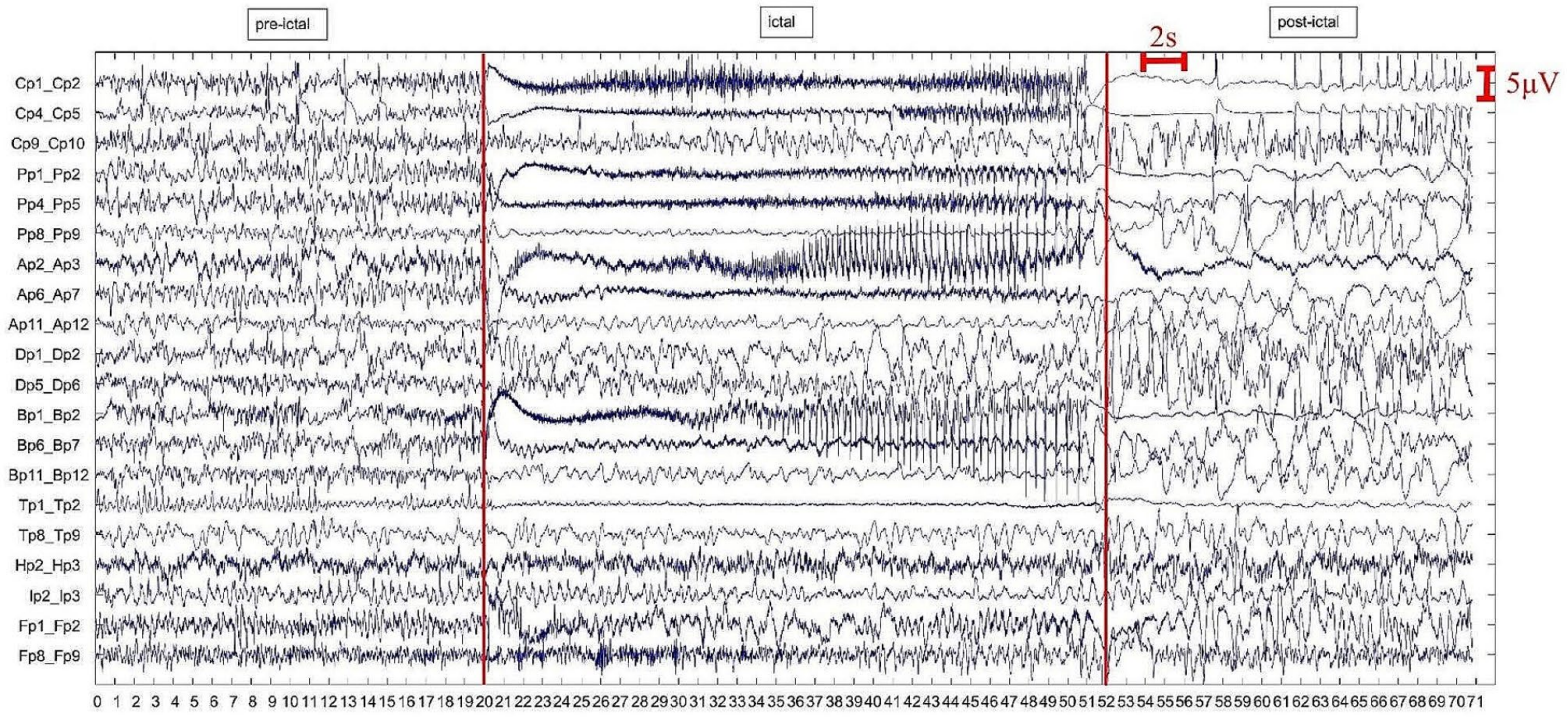
iEEG recording for seizure 1. It has a total length of 72s and seizure onset of up to 32 s (20s ~ 52s). Each channel corresponds to a bipolar iEEG signal, and the associated two sensor names are recorded on the vertical axis. The two red vertical lines divide the data into three phrases: pre-ictal, ictal, and post-ictal phases. The horizontal axis represents time

The experimental database consisted of 72-second or 64-second iEEG signals, which were recorded using invasive electrodes equipped with 20 channels and placed in specific areas of the cerebral cortex, and the sampling frequency was 256 Hz. The positions of electrode insertion were determined based on preliminary clinical and electrophysiological examinations. In addition, iEEG signals were bipolar, that is, obtained as the difference in potentials recorded on two adjacent sensors. For example, the first channel in Fig. 1 (the top channel) is named Cp1, which means that the value of this channel is the potential of sensor Cp1 minus the potential of sensor Cp2. For convenience, the name of the first channel is denoted afterwards as Cp1, the name of the second channel is denoted as Cp4, and so on.

As shown in Fig. 1, each signal is divided into three phases: pre-ictal, ictal, and post-ictal phases. According to clinical experts, the ictal phrase which corresponds to the epileptic seizure onset can also be divided into three overlapped epochs (named ictal 1, ictal 2 and ictal 3, respectively). Figure 1 shows only the iEEG signal of seizure 1. The iEEG acquisition method, channel naming method, and phrase division method of seizure 2 and seizure 3 are the same as those of seizure 1. The difference is that the signal length of seizure 1 is 72 s, and the signal lengths of seizure 2 and seizure 3 are 64 s (see Table 1). It is worth noting that the ictal phase in seizure 1 lasted 32 s (20s ~ 52s), the ictal phase in seizure 2 and seizure 3 lasted 24 s (20s ~ 44s). Table 2 shows the start time and end time of each epoch of seizure 1, Table 3 is for seizure 2 and seizure 3. As shown in Table 2, there is a 4-second interval before and after the ictal phase, because it is hard to clearly define the exact time point at which epilepsy begins and ends during this period and remove this part of the data can increase the credibility of experimental results. However, there is no theoretical support how to determine the length of this ambiguous time period. In this study, this time period for seizure 1 was identified as 4 s, and seizure 2 and seizure 3 were identified as 2 s. In addition, 2 s of data at the beginning (0–2 s) and end (70–72 s) of the data are removed. For the stability of the experimental results and the smooth start of the RNN-GC method, we set the signal length of each epoch to 16 s, and there is 10 s of data overlap between ictal 1, ictal 2, and ictal 3 of seizure 1 (see Table 2). The overlap length of the three epochs of seizure 2 and seizure 3 is set to 12 s (see Table 3). The overlapped data is all part of the ictal data, so the difference in the overlapped length will not have a great impact.

**Table 2:** The beginning point and ending point of each epoch of seizure 1

Epoch name	Start point [s] ~ End point [s]	Length [s]
pre-ictal	2 ~ 18	16
ictal 1	22 ~ 38	16
ictal 2	28 ~ 44	16
ictal 3	34 ~ 50	16
post-ictal	54 ~ 70	16

**Table 3:** The beginning point and ending point of each epoch of seizure 2 and seizure 3

Epoch name	Start point [s] ~ End point [s]	Length [s]
pre-ictal	2 ~ 18	16
ictal 1	20 ~ 36	16
ictal 2	24 ~ 40	16
ictal 3	28 ~ 44	16
post-ictal	46 ~ 62	16

According to the preliminary analysis of clinical experts, the 20 channels can be categorized into three groups according to their involvement along the different phases or epochs of the seizure (see Table 4). These three groups are named Group O (Onset Group), Group P (Propagation Group) and Group N (Not-involved Group). Signals associated with Group O concern the main area of the epileptic seizure or the source area of the epilepsy, which has a great correlation with the seizure onset. Signals belonging to Group P are abnormal electrical signals that produced in normal brain area due to the influence of signal transmission in the seizure onset. The signals of Group N are considered to be unaffected by the seizure, that is, they do not participate in the construction of the seizure propagation networks.

**Table 4:** Categories of the channels in the real iEEG

Group name	Channels name	Total channels
O (Onset)	Cp1, Cp4, Pp1, Pp4, Ap2, Ap6, Bp1	7
P (Propagation)	Pp8, Dp1, Dp5, Tp1, Fp2	5
N (Not-involved)	Cp9, Ap11, Bp6, Bp11, Tp8, Hp2, Ip2, Fp8	8

Since effective connectivity is mainly used to infer the direction of information flow between brain regions, and our purpose is to locate the brain region that causes seizures, we mainly study the direction of information flow in each channel in Group O and Group P and we did not care about Group N, which was not involved in seizures.

### Recurrent neural network-based granger causality (RNN-GC)

Recurrent neural network uses cycled connections in units to remember dynamic temporal activity throughout history. Classical RNN models usually suffer from the vanishing gradient when trained with backpropagation through time [27], and this problem becomes more serious as the layers of the network become deeper, and Bengio et al. [28] found some pretty fundamental reasons why RNNs are not able to handle such “long-term dependencies”. To solve this problem, long short-term memory network with memory cell units [29] has been introduced. Many variants of LSTM networks have been proposed, and a slightly more dramatic variation on the LSTM is the Gated Recurrent Unit (GRU), introduced by Cho et al. [30]. It combines the forget and input gates into a single “update gate”, and it also merges the cell state and hidden state. The resulting model is simpler than standard LSTM models, and has been growing increasingly popular. Greff et al. [31] have done a nice comparison of popular variants, and found that certain modifications, such as coupling the input and forget gates or removing peephole connections, which have not only decreased performance, but also reduced the number of parameters and computational costs of the LSTM. Therefore, GRU is easier to converge, and the risk of overfitting is smaller when the data set is smaller. In this work, we use GRU as the recurrent network unit of the RNN-GC model instead of LSTM as mentioned in [24], and call it GRU-GC model.

**Fig. 2:**
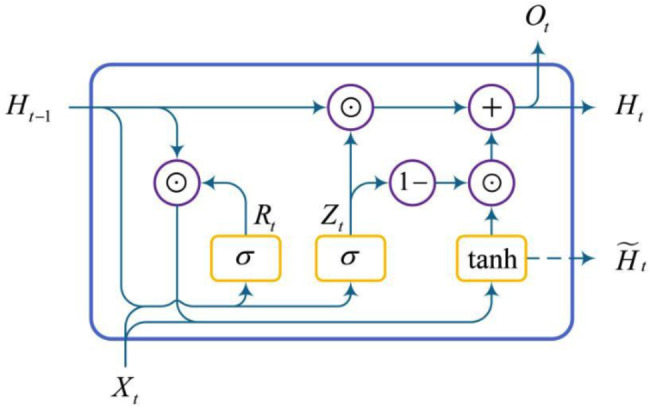
GRU structure diagram. Each line carries an entire vector, from the output of one node to the inputs of others. The purple circles represent the various operations of the vector, like vector addition, vector dot product. The yellow boxes are activation functions. Lines merging denote concatenation, and a line forking denote its content being copied and the copies going to different locations

GRU introduces a gating mechanism to better capture long-term dependencies in time series. The GRU includes two gates, a reset gate and an update gate (see Fig. 2). The gating mechanism is used to determine when to update the hidden state and when to reset it. For instance, a reset gate would allow to control how much of the previous state we might still need to remember. Likewise, an update gate would allow to control how much of the new state is just a copy of the old state. Reset gate and update gate are both vectors with entries in (0, 1).

The inputs of the reset gate and update gate of the GRU are both the current time step input ${X_t}$ and the hidden state ${H_{t - 1}}$ of the previous time step, and the output is calculated by the fully connected layer whose activation function is a sigmoid $\sigma$. Assuming that the number of hidden units of the GRU is $h$, the number of samples is $n$, and the dimension of the input signal is $d$, for a given time step $t$, the reset gate${R_t} \in {{\mathbb{R}}^{n \times h}}$and update gate${Z_t} \in {{\mathbb{R}}^{n \times h}}$can be calculated by Eq. 1.

1 $$\begin{gathered} {R_t}=\sigma \left( {{X_t}{W_{xr}}+{H_{t - 1}}{W_{hr}}+{e_r}} \right) \hfill \\ {Z_t}=\sigma \left( {{X_t}{W_{xz}}+{H_{t - 1}}{W_{hz}}+{e_z}} \right) \hfill \\ \end{gathered}$$

where ${W_{xr}},{W_{xz}} \in {{\mathbb{R}}^{d \times h}},{W_{hr}},{W_{hz}} \in {{\mathbb{R}}^{h \times h}}$ are weight parameters and ${e_r},{e_z} \in {{\mathbb{R}}^{1 \times h}}$ are biases. To update the hidden state, we need to calculate the candidate hidden state${\widetilde {H}_t} \in {{\mathbb{R}}^{n \times h}}$, which is defined as:

2 $${\widetilde {H}_t}=\tanh \left( {{X_t}{W_{xh}}+\left( {{R_t} \odot {H_{t - 1}}} \right){W_{hh}}+{e_h}} \right)$$

From Eq. 2 it can be seen that the reset gate controls how the hidden state of the previous time step flows into the candidate hidden state of the current time step. The hidden state of the previous time step may contain all the historical information of the time series up to the previous time step. Therefore, the reset gate can be used to discard historical information that is not related to the prediction and help capture short-term dependencies in the time series. The calculation formula for the hidden state ${H_t} \in {{\mathbb{R}}^{n \times h}}$is defined as:

3 $${H_t}={Z_t} \odot {H_{t - 1}}+\left( {1 - {Z_t}} \right) \odot {\widetilde {H}_t}$$

when the update gate ${Z_t}$ is close to 1, the old state is retained, then the information from ${X_t}$ will be ignored, thereby effectively skipping the time step $t$ in the dependency chain. Conversely, when ${Z_t}$ approaches 0, the new hidden state ${H_t}$ will approach the candidate hidden state ${\widetilde {H}_t}$.Therefore, the update gate helps in capturing long-term dependencies in time series.

The GRU-GC model still uses the method mentioned in [24] to calculate WGCI. The causal matrix calculated by GRU-GC model is denoted as $G=\left\{ {{c_{ij}}} \right\} \in {{\mathbb{R}}^{m \times m}}$:

4 $$G = \left[ {\begin{array}{*{20}{c}}{{c_{11}}} & {{c_{12}}} & \cdots & {{c_{1m}}} \\ {{c_{21}}}&{{c_{22}}}& \cdots &{{c_{2m}}} \\ \vdots & \vdots & \ddots & \vdots \\ {{c_{m1}}}&{{c_{m2}}}& \cdots &{{c_{mm}}} \end{array}} \right]$$

where $m$ represents the dimension of the signal, ${c_{ij}}$ represents the causal effect of signal $i$ onto signal $j$. ${c_{i * }}$ represents the causal effect of signal $i$ onto other signals, excluding the effect of the signal itself.

In order to make the cause and effect of signal $i$ comparable to other signals, we normalize it:

5 $$c_{{ij}}^{ * }=\frac{{{c_{ij}}}}{{\sum\nolimits_{{j=1}}^{m} {{c_{ij}}} }}$$

Then, we binarize the causal matrix, and the threshold is ${\phi _G} \in \left( {0,1} \right)$, the resulting new matrix is represented by $\widetilde {G}$:

6 $$\tilde G = \left[ {\begin{array}{*{20}{c}}{\widetilde {{c_{11}}}}&{\widetilde {{c_{12}}}}& \cdots &{\widetilde {{c_{1m}}}} \\ {\widetilde {{c_{21}}}}&{\widetilde {{c_{22}}}}& \cdots &{\widetilde {{c_{2m}}}} \\ \vdots & \vdots & \ddots & \vdots \\ {\widetilde {{c_{m1}}}}&{\widetilde {{c_{m2}}}}& \cdots &{\widetilde {{c_{mm}}}} \end{array}} \right]$$

where the element $\widetilde {{{c_{ij}}}}$ in $\widetilde {G}$ is defined as:$\widetilde {{{c_{ij}}}}=0, c_{{ij}}^{ * }<{\phi _G}$, otherwise $\widetilde {{{c_{ij}}}}=1, c_{{ij}}^{ * } \geqslant {\phi _G}$.

### Group analysis

In recent years, the development of quantitative analysis of complex networks based on graph theory has been rapidly applied to the study of brain network. The structural and functional of the brain are characterized by complex networks, such as small-world topologies, highly connected hubs, and modularity, both at the whole-brain scale of human neuroimaging and at the cellular scale of non-human animals [32]. The brain tends to follow two basic principles of functional organization: functional segregation and functional integration [26]. Functional segregation means that specific areas perform specific functions, and functional integration means that specific tasks require dynamic information exchange and interaction between different areas. Distributed information transfer in local regions of the brain can be described by a network model, including a set of nodes (neurons, regions) and edges (interregional connections, pathways) [26]. Understanding functional segregation and functional integration between different regions of the brain is critical in decoding the mechanisms of normal physiological brain activity and brain disorders, especially epilepsy [33]. Graphical models provide means to characterize complex brain connectivity networks, so-called brain graphs [34]. In this study, each iEEG channel corresponds to a region of the brain. We can consider the region corresponding to a single channel as a node, and the effective connectivity between regions as edges, thereby forming a directed graph model. Based on the graph model, we can perform group analysis and centrality analysis (see Eqs. (6–9)) on iEEG data.

**Fig. 3:**
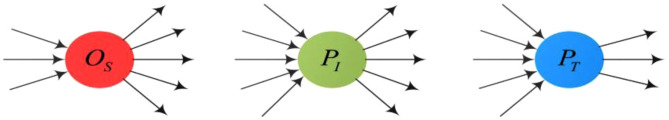
Three types of nodes. The red circle indicates a node belonging to the Onset Source group (${O_S}$), which has $d_{i}^{{(in)}} \leqslant d_{i}^{{(out)}}$, the green circle indicates a node belonging to the Propagation Internal group (${P_I}$) which has $d_{i}^{{(in)}} \approx d_{i}^{{(out)}}$and the blue circle indicates a node belonging to the Propagation Sink group (${P_T}$) which has $d_{i}^{{(in)}} \gg d_{i}^{{(out)}}$. The arrow represents the direction of the signal flowing through the node

The adjacency matrix of the graph model is $\tilde {G}$, from which the out-degree $d_{i}^{{(out)}}$ and in-degree $d_{i}^{{(in)}}$ of each node can be calculated, and the nodes are classified into three types: ${O_S}$ (Onset Source group), ${P_I}$ (Propagation Internal group), ${P_T}$ (Propagation Target node) according to the difference between the in-degree and out-degree (see Fig. 3).

Define the degree centrality [35] (Eq. 6) and the in-out degree (Eq. 7) of the directed connected graph as:

7 $$d{c_i}=\frac{{d_{i}^{{(in)}} - d_{i}^{{(out)}}}}{{d_{i}^{{(in)}}+d_{i}^{{(out)}}}}$$

8 $$\left\{ \begin{gathered} d_{i}^{{(in)}}=\sum\limits_{{j \in \left[ {1,m} \right],j \ne i}} {{{\widetilde {G}}_{ji}}} \hfill \\ d_{i}^{{(out)}}=\sum\limits_{{j \in \left[ {1,m} \right],j \ne i}} {{{\widetilde {G}}_{ij}}} \hfill \\ \end{gathered} \right.$$

To classify the nodes, a threshold, ${\phi _{dc}} \in \left( {0,1} \right)$, is used. The node $i$ is classified in the following way:

9 $$i \in \left\{ \begin{gathered} {O_S}, d{c_i} \leqslant - {\phi _{dc}} \hfill \\ {P_I}, - {\phi _{dc}}<d{c_i}<{\phi _{dc}} \hfill \\ {P_T}, d{c_i} \geqslant {\phi _{dc}} \hfill \\ \end{gathered} \right.$$

A low value of ${\phi _{dc}}$ results in more elements in ${O_S}$ and ${P_T}$, and vice versa.

### Centrality analysis

Centrality is one of the core principles of network or graph analysis which measures how “central” a node is, and estimates the importance of a node in the network. However, depending on the application and perspective, what counts as “central” may vary depending on the context. Correspondingly, there are a number of ways to measure centrality of a node. In this work, the betweenness centrality [36, 37] and PageRank centrality [38] are considered to study the epileptic seizure graph model.

Betweenness centrality measures how important a node is to the shortest paths through the network. The betweenness of a specific node is equal to the number of shortest paths from all pairs of nodes in the graph that pass through that node. Informally, the more the shortest paths that go through a node, the more important that node is in terms of graph connectivity. That is, the higher the betweenness centrality value, the more central the node is. Formally, the betweenness centrality of node $i$ is:

10 $$b{c_i}=\sum\limits_{{j,k \in \left[ {1,m} \right],j \ne k}} {\frac{{n_{{jk}}^{{(i)}}}}{{{n_{jk}}}}}$$

where ${n_{jk}}$ is the number of shortest paths between node $j$ and $k$, and $n_{{jk}}^{{\left( i \right)}}$ is the number of shortest paths between node $j$ and $k$ that pass through node $i$. The sum in the expression ranges over all pairs of distinct vertices $j$ and $k$.

The PageRank can be considered as the “importance score” of a network node. This importance score will always be a non-negative real number and all the scores will add to 1. The core idea of PageRank centrality is to start from any node, randomly walk towards the nodes it connects, and then continue to walk repeatedly, and finally calculate the probability based on the number of visits to each node. This probability is the value of PageRank centrality. The definition here is:

11 $$p{r_{i|t+1}}=\frac{{1 - \lambda }}{m}+\lambda \sum\limits_{{j \to i}} {\frac{{p{r_{j|t}}}}{{d_{j}^{{(out)}}}}}$$

where $\lambda$ represents the damping coefficient, and the general value is 0.85, and Eq. 10 can also be expressed in matrix form:

12 $$P{R_{t+1}}=\lambda M \bullet P{R_t}+\frac{{1 - \lambda }}{m}$$

where $P{R_{t+1}} \in {{\mathbb{R}}^{m \times 1}}$ represents the PageRank value of the directed connected graph at $t+1$ iterations, $M$ is the weighted adjacency matrix of the connected graph ($\sum\nolimits_{{i=1}}^{m} {{M_{ij}}} =1$), and the weight represents the probability that node $j$ is connected to node $i$. The calculation method of $M$ is shown in Eq. 12. In order to represent the probability distribution, we use the function $f$ (Eq. 13) to normalize the data:

13 $$M=f({G^T} \bullet {\tilde {G}^T})$$

14 $$f\left( x \right){\text{ = }}\left\{ \begin{gathered}0, {\left\| x \right\|_1} = 0 \hfill \\\frac{x}{{{{\left\| x \right\|}_1}}}, {\left\| x \right\|_1} \ne 0 \hfill \\ \end{gathered} \right.$$

The betweenness centrality and PageRank centrality are used to analyse the importance of each node in the graph model of seizures, and provide a basis for lesion localization.

## Results

### Experiments and parameters setting

The experiment uses the RNN-GC [24] model to analyse the intracranial EEG signals of an epileptic patient and GRU is the basic model of the recurrent network (GRU-GC). The parameter settings of the model are listed in Table 5. During the GRU-GC model training process, we used the AdBound optimizer [39] to optimize the loss function. According to the definition of WGCI: in order to obtain the prediction error for a certain variable, we need to use different kinds of variables for regression. Therefore, the loss function used by the model is the mean squared error (MSE). To avoid overfitting the model, dropout parameters are set to 0.5 in the model, and the dimensions of the GRU hidden layer and the number of epochs are set relatively small, 30 and 10 respectively.

**Table 5:** Value setting and meaning of model parameters

Parameter name	Value	Parameter meaning
num_layers	1	Number of layers of the recurrent network.
dropout	0.5	Probability of randomly removing some neurons.
lr_rate	0.001	Learning rate of optimizer.
momentum	0.9	Momentum of optimizer.
weight_decay	0.001	Weight decay of optimizer.
bt_sz	32	Batch size for training.
seq_len	50	Sequence length of GRU input.
hidden_dim	30	Dimension of GRU hidden layer.
num_epoch	10	Training epoch.
${\phi _G}$	0.1	Threshold of binarization of connected matrix.
${\phi _{dc}}$	0.1	Threshold for classifying nodes.
$\lambda$	0.85	PageRank’s Damping Coefficient.

The data used in the experiment was the intracranial EEG from three seizures of the same epileptic patient. We used the GRU-GC model to perform connectivity analysis, group analysis, and centrality analysis on these three groups of data. In order to make the results of the experiment more credible, we performed 10 experiments with the same settings on the same set of data, and took the average value as the final result. After training the GRU-GC model, we get the connectivity matrix corresponding to seizure 1, seizure 2, seizure 3. The element ${c_{ij}}$ in the matrix represents the strength of the connectivity between node $i$ and node $j$, and the direction of connectivity is from node $i$ to node $j$. Then, according to the threshold ${\phi _G}$ the connectivity matrix is calculated to get the binary connectivity matrix which indicates whether the two nodes are connected or not, and the directed connectivity of different epochs of the seizures can be drawn accordingly. This binary matrix is called graph adjacency matrix in graph theory, and group analysis and centrality analysis are also calculated on the basis of adjacency matrix.

### Evaluation metrics

The experiments were performed on a computer with an Intel i5-8600 CPU and a NVIDIA GeForce 1070Ti GPU. The computer was Windows 10 system and the model was implemented using the PyTorch framework. The intracranial EEG data used in the experiment included a total of 20 channels (nodes). According to the clinician, the 20 channels were divided into three groups O, P, and N (see Table 4). The intracranial EEG data used in the experiment included a total of 12 channels (excluding the N group), of which 7 channels were in the O group and 5 channels were in the P group. According to the degree centrality of the adjacency matrix, we divide the nodes into three categories: ${O_S}$, ${P_I}$, ${P_T}$. The out-degree and in-degree of a ${P_I}$-type node is roughly equivalent, and it is impossible to accurately determine whether it belongs to Group O or Group P. Therefore, the nodes in the ${P_I}$ group belong to the O group or the P group determined by the expert.

Here, we use classification accuracy to evaluate the performance of group analysis, including Acc1 and Acc2. Acc1 classifies ${P_I}$ as O group, Acc2 classifies ${P_I}$ as P group, and at the same time, we mark the results of each channel (node) group analysis are consistent with the results of expert classification. “T” is used to indicate that the two results are consistent or correctly classified, “F” for misclassification, and “M” indicates the result of the group analysis is ${P_I}$.

## Results

**Fig. 4:**
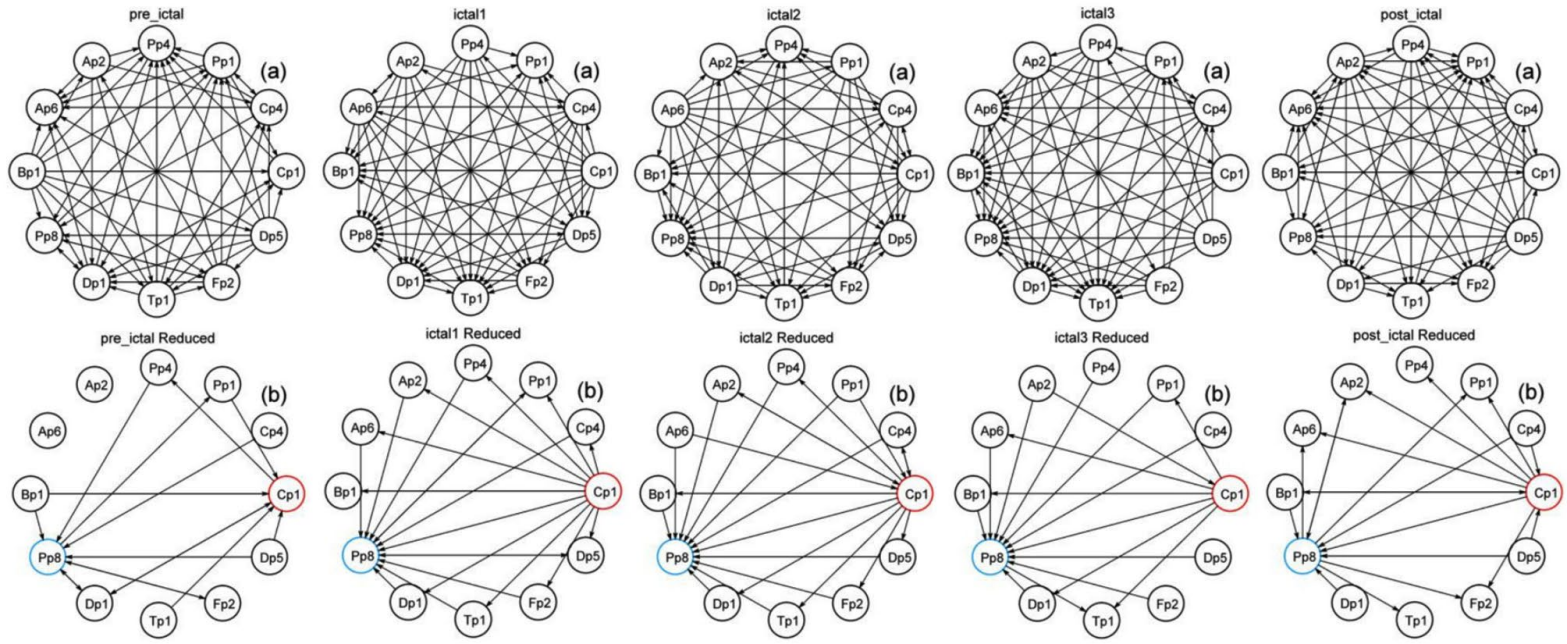
Directed connected graphs for each epoch of the seizure 1. Each epoch contains (**a**) (**b**) two subgraphs. (**a**) represents the connection between all nodes, and (**b**) represents a simplified version of the connected graph. The sub-graph (**b**) only shows the connections related to the red and blue nodes, including the connections starting from and arriving at these two nodes. The red nodes belong to group , and the blue nodes belong to group . Arrows indicate the direction of information flow

Figure 4 shows the directed connected graph of each stage of seizure 1, and the connected graphs of seizure 2 and seizure 3 can also be drawn using the same method. The directed connected graph is drawn according to the adjacency matrix calculated by the GRU-GC algorithm. The nodes correspond to the 12 channels of intracranial EEG signals, and the edges represent the information flow between the nodes.

Tables 6 and 7 are the classification results of nodes by group analysis. At the same time, we also calculated the matching degree between the result and the expert’s result, that is, the classification accuracy rate (represented by fractions). Table 6 is the statistics of group O, and Table 7 is the statistics of group P.

**Table 6:** Group analysis results and accuracy statistics of each epoch of seizure 1, seizure 2, and seizure 3 (Group O only)

Seizures	Epoch	Group O							Acc1	Acc2
		Cp1	Cp4	Pp1	Pp4	Ap2	Ap6	Bp1		
seizure 1	pre-ictal	F	M	M	F	T	F	T	2/7	4/7
	ictal 1	T	T	F	T	T	T	F	5/7	5/7
	ictal 2	T	T	T	F	T	T	F	5/7	5/7
	ictal 3	T	T	T	T	T	F	F	5/7	5/7
	post-ictal	T	T	F	T	T	F	T	5/7	5/7
seizure 2	pre-ictal	F	T	T	M	F	F	F	2/7	3/7
	ictal 1	T	T	T	T	M	F	M	4/7	6/7
	ictal 2	T	M	T	F	T	T	T	5/7	6/7
	ictal 3	T	T	T	F	M	T	T	5/7	6/7
	post-ictal	F	T	F	F	T	F	F	2/7	2/7
seizure 3	pre-ictal	T	T	F	T	F	M	F	3/7	4/7
	ictal 1	T	T	T	T	F	F	F	4/7	4/7
	ictal 2	T	F	T	T	T	T	T	6/7	6/7
	ictal 3	T	T	T	T	F	F	T	5/7	5/7
	post-ictal	F	M	T	M	M	F	F	1/7	4/7

**Table 7:** Group analysis results and accuracy statistics of each epoch of seizure 1, seizure 2, and seizure 3 (Group P only)

Seizures	Epoch	Group *P*					Acc1	Acc2
		Pp8	Tp1	Fp2	Dp1	Dp5		
seizure 1	pre-ictal	T	F	F	T	F	2/5	2/5
	ictal 1	T	T	T	T	M	4/5	5/5
	ictal 2	T	T	T	F	T	4/5	4/5
	ictal 3	T	T	F	T	F	3/5	3/5
	post-ictal	M	T	T	F	F	2/5	3/5
seizure 2	pre-ictal	T	F	F	M	F	1/5	2/5
	ictal 1	T	T	F	T	F	3/5	3/5
	ictal 2	T	T	T	F	T	4/5	4/5
	ictal 3	T	T	T	T	F	4/5	4/5
	post-ictal	T	F	F	F	F	1/5	1/5
seizure 3	pre-ictal	F	T	T	T	F	3/5	3/5
	ictal 1	T	T	T	M	F	3/5	4/5
	ictal 2	T	T	T	T	T	5/5	5/5
	ictal 3	T	T	F	T	F	3/5	3/5
	post-ictal	T	F	F	T	F	2/5	2/5

**Fig. 5:**
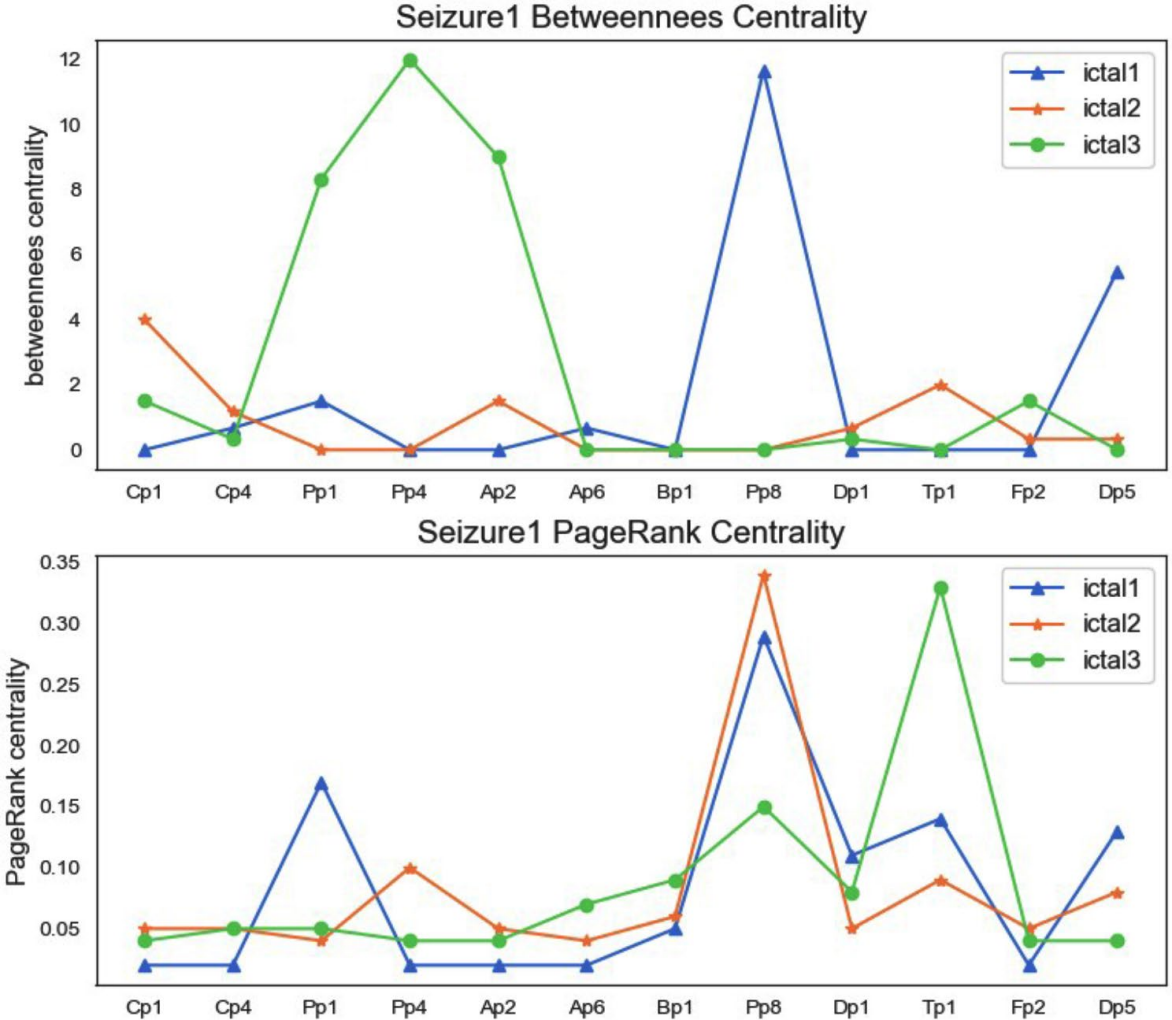
Betweenness centrality and PageRank centrality in seizure1 ictal phrase (ictal 1, ictal 2, and ictal 3)

**Fig. 6:**
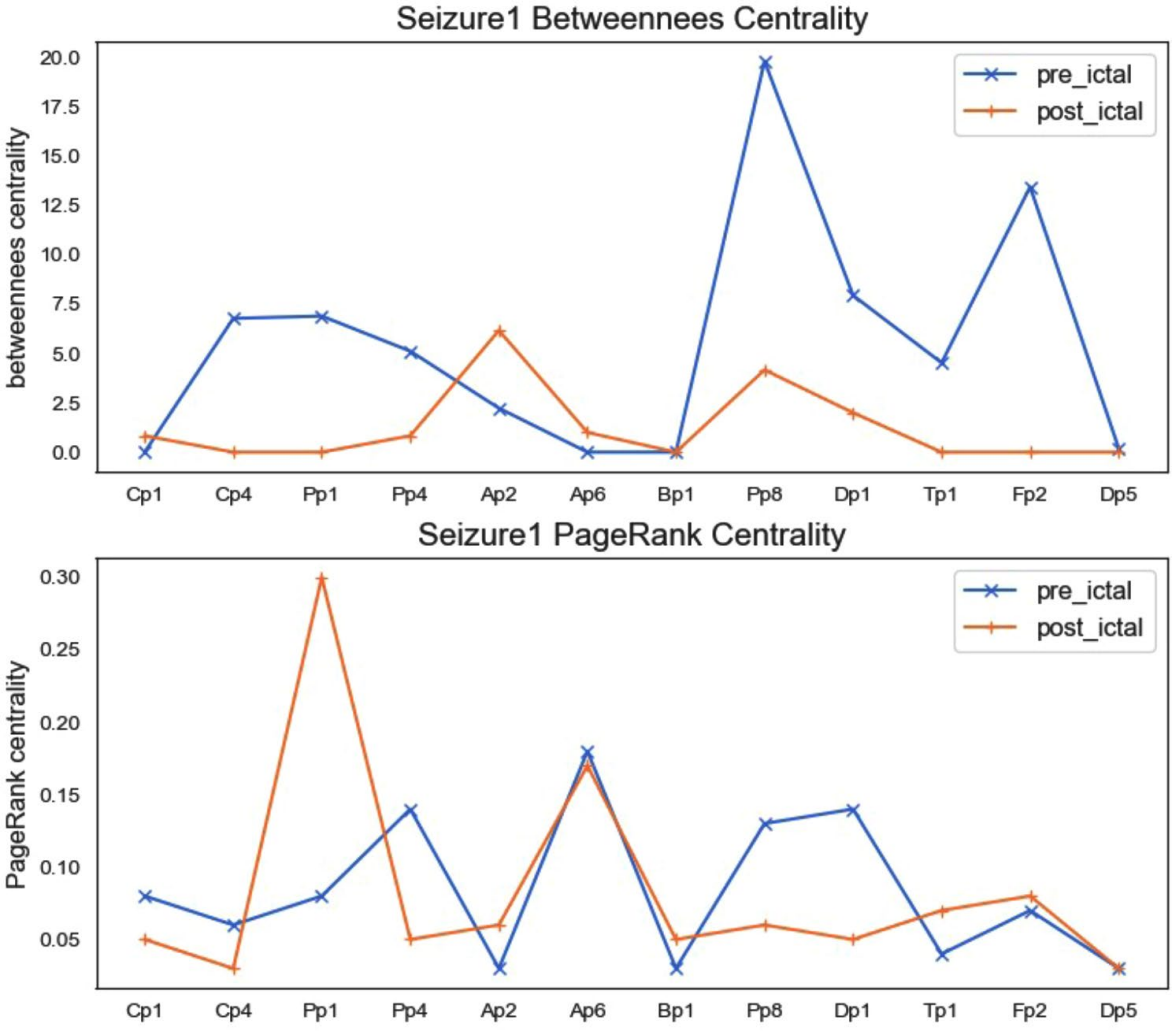
Betweenness centrality and PageRank centrality in seizure1 pre-ictal and post-ictal phrase

Figures 5 and 6 are line charts of the centrality analysis of each node, and they are only the results of seizure 1, and the results of seizure 2 and seizure 3 can also be obtained by the same method. Each graph contains two subgraphs. The upper graph represents the betweenness centrality of each node, and the lower graph represents the PageRank centrality. We separate the centrality result of the ictal phase because the seizure phase (ictal1, ictal2, ictal3) is different from the connectivity and centrality before and after the seizure.

## Discussion

In this work, our task was to study the temporal evolution pattern of brain effective connectivity in epileptic patients and the classification accuracy of group analysis based on GRU-GC model. Therefore, we discuss the experimental results from two aspects: performance analysis and connectivity analysis.

### Performance analysis

From Tables 6 and 7, it is concluded that no matter it is seizure 1, seizure 2, and seizure 3, the group analysis based on the GRU-GC algorithm performs well in the ictal phrase. The expert classification is also for the seizure (ictal) phase, not for pre-ictal and post-ictal phase. In the opinion, group O has a total of 7 channels, and group P has a total of 5 channels. For the classification of group O by our algorithm, the classification accuracy can best reach 6/7 and 5/7 with and without ${P_I}$ included. For the classification of group P, the classification accuracy can best reach 4/5 and 3/5 with and without ${P_I}$ included. Further observation revealed that the classification accuracy of ictal 1, ictal 2 and ictal 3 was very high, that is, the function of the brain related areas did not change significantly during the whole course of the seizure. Seizure 1, seizure 2, and seizure 3 intracranial EEG signals were recorded from the same patient for three seizures, and the results were consistent to provide more evidence for our analysis.

For a period of time before and after the ictal stage, the coupling effect between the signals was not obvious, and clinical experts were unable to give an accurate classification opinion. In the experiment, we still set the ground truth of pre-ictal and post-ictal to be the same as the expert opinion. From the observation and comparison of Tables 6 and 7, we also found that the classification effect of pre-ictal and post-ictal is not satisfactory. It also shows that certain areas of the brain are affected by seizures.

### Connectivity analysis

After observing and comparing from Fig. 4; Table 6, and Table 7, we found that the connectivity of the brain is different among the seizure stages (pre-ictal, ictal and post-ictal), and the whole process of the seizure(ictal1,ictal2,ictal3) remains same. The analysis from Figs. 5 and 6 shows that centrality also conforms to this conclusion.

Betweenness centrality is defined as the ratio between the number of shortest paths through a particular node and the total number of shortest paths in the network. That is, nodes with high betweenness centrality play an important role in the entire network, because a large number of shortest paths in the network pass through this important node. Therefore, once we remove this node, large-scale structural changes will occur in the entire network. The implication for epilepsy surgery is that when processing a node with a high betweenness centrality (corresponding to a certain region of the brain), the resection is only performed with a high degree of confidence, because this node region cannot be completely determined pathology and whether it involves the connection of functional areas of the brain, care should be taken, once the resection may cause the loss of normal brain function.

According to the definition of PageRank centrality: the larger the PageRank value, the greater the probability that the node is ${P_T}$ type. The smaller the PageRank value, the greater the probability that the node is ${O_S}$ type. Therefore, for the channels of Cp1, Cp4, Pp1, Pp4, Ap2, Ap6, and Bp1, the PageRank index should be relatively small, and for the channels of Pp8, Dp1, Dp5, Tp1, and Fp2, the PageRank index should be relatively large. From the visual analysis of PageRank centrality in Figs. 5 and 6, we can see that the experimental results basically meet this ideal result.

## Conclusions

In this study, we mainly studied the classification and connection mode of various channels in seizures, using data from intracranial EEG signals recorded from multiple seizures in the same epileptic patient. Our experiments found that effect connectivity remained relatively stable throughout the seizure, but was different before and after the seizure. At the same time, it was found that some nodes have high centrality and play an important role in the seizure network. Before surgery, it should be fully clear whether the corresponding areas of these nodes are brain functional areas. The results of the group analysis at the ictal stage are highly consistent with the classification results suggested by clinical experts, which fully illustrates the effectiveness of our method. Group analysis provides theoretical guidance for epilepsy localization, and is an effective auxiliary method for preoperative evaluation. The results of centrality and connectivity analysis also provide some basis for revealing the evolutionary pattern of functions between various regions of the brain during seizures.

## Data Availability

No datasets were generated or analysed during the current study.
